# Principles for managing OUD related to chronic pain in the Nordic countries based on a structured assessment of current practice

**DOI:** 10.1186/s13011-018-0160-7

**Published:** 2018-06-01

**Authors:** Johan Kakko, Charlotte Gedeon, Mikael Sandell, Henrik Grelz, Inge Birkemose, Thomas Clausen, Valgerður Rúnarsdóttir, Kaarlo Simojoki, Richard Littlewood, Hannu Alho, Fred Nyberg

**Affiliations:** 1 Department of Clinical Sciences, Psychiatry, Umeå University, Psykiatriska Kliniken Umeå, Norrlands Universitetssjukhus, SE-901 85 Umeå, Sweden; 2 Solstenen i Skane, Addiction Centre, Lund, Sweden; 3 Capio Maria, Stockholm and Skåne, Sweden; 4 Department of Clinical Sciences Lund University, Malmö, Sweden; 5 Pain Rehabilitation Department, Skåne University Hospital, Skåne, Sweden; 6 Overlæge, Odense Kommune, Misbrugsbehandling, Odense, Denmark; 7 Norwegian Centre for Addiction Research, University of Oslo, Oslo, Norway; 8 SAA – National Center of Addiction Medicine, Vogur Hospital, Reykjavik, Iceland; 9 A-Clinic Foundation/ A-clinic oy, University of Helsinki and Helsinki University Hospital, Helsinki, Finland; 10 Applied strategic, London, UK; 11 Abdominal Center, University Hospital and University of Helsinki, Helsinki, Finland; 12 Department of Pharmaceutical Biosciences, Uppsala University, Uppsala, Sweden

**Keywords:** Opioid use disorder, Chronic pain, Nordics countries

## Abstract

**Background:**

Long-term use of opioid analgesics (OA) for chronic pain may result in opioid use disorder (OUD). This is associated with adverse outcomes for individuals, families and society. Treatment needs of people with OUD related to chronic pain are different compared to dependence related to use, and also injection, of illicit opioids. In Nordic countries, day-to-day practical advice to assist clinical decision-making is insufficient.

**Aim:**

To develop principles based on expert clinical insights for treatment of OUD related to the long-term use of OA in the context of chronic pain.

**Methods:**

Current status including an assessment of barriers to effective treatment in Finland, Denmark, Iceland, Norway, Sweden was defined using a patient pathway model. Evidence to describe best practice was identified from published literature, clinical guidelines and expert recommendations from practice experience.

**Results:**

Availability of national treatment guidelines for OUD related to chronic pain is limited across the Nordics. Important barriers to effective care identified: patients unlikely to present for help, healthcare system set up limits success, diagnosis tools not used, referral pathways unclear and treatment choices not elucidated. Principles include the development of a specific treatment pathway, awareness/ education programs for teams in primary care, guidance on use of diagnostic tools and a flexible treatment plan to encourage best practice in referral, treatment assessment, choice and ongoing management via an integrated care pathway. Healthcare systems and registries in Nordic countries offer an opportunity to further research and identify population risks and solutions.

**Conclusions:**

There is an opportunity to improve outcomes for patients with OUD related to chronic pain by developing and introducing care pathways tailored to specific needs of the population.

## Background

The long-term use of opioid analgesics (OA) in the context of chronic pain syndromes may result in opioid use disorder (OUD) [1]. This is associated with harm to individuals, their families and also to society [2]. OUD in this population ranges from mild disease with few criteria of OUD fulfilled to severe and represents a heterogeneous population [3]. For the patient, each dose will provide some immediate relief, but repeated use of opioids can worsen pain, and associated psychological symptoms. Dependence, when established, interacts with pain and associated symptoms such as sleep-disturbance, psychological distress, tiredness and cognitive symptoms. This may result in a perceived need for opioids or other medication for control of such symptoms [4]. Chronic pain may alone interfere severely with the ability to participate in work and social life; the impact of long-term opioid therapy at the individual level is unclear. Long-term opioid therapy, often considered in excess of 3 months [5, 6], has a dose-dependent relation to the development of OUD and consistent use of high doses may indicate risk of the problem [7].

Without intervention, OUD commonly results in serious psychosocial issues, medical problems and a significant risk from overdose [8]. Pharmacological choices indicated for the treatment of OUD in this population include tapering of OA or maintenance therapy with opioid agonist therapy (OAT) such as buprenorphine/ naloxone or buprenorphine [9, 10] and methadone [11, 12]. Prescription of OAT for OUD must be considered as distinct from use of opioids for pain, even though products may have use in both situations.

It is recognised that the characteristics [13], behaviours [14] and needs [15] of patients with OUD related to chronic pain are often different when compared to patients with dependence related to use and also injection of illicit opioids [12]. OUD may often be in the context of the use of many sources and types of opioid especially when severe, however when related to chronic pain distinct features are present. Problems such as social disadvantage, contact with the criminal justice system, and co-existent health problems may be less common in those with OUD related to chronic pain and prescribed OA [12], especially during early phases of the problem. Illicit heroin is now not the only source of opioid and in some countries, is not the major problem: OA may make up a large proportion of the opioid problem whether prescribed, illicit or obtained from family or other contacts [16]. These differences point to the potential for early intervention and the need for a specific treatment approach tailored to the requirements of this population [12].

Countries have published guidelines for the use of OA in relation to chronic pain (e.g. USA [17], UK [18], Australia [19]) and for the management of OUD in general [20]. Specific guidance for OUD related to chronic pain is only available in a limited number of countries (e.g. UK [21], Spain [22], Australia [23]) and does not aid the set-up of care services or support practical, day-to-day decisions in management: the problem has not been addressed optimally in any country so far. In the Nordics common approaches to the management of OUD and access to healthcare is an opportunity to assess clinical practice. This work aims to assess current approaches, limits and successes for care of patients with OUD related to chronic pain and, based on this analysis, outline principles for policy and practice development in the Nordic countries to improve outcomes for patients [12].

## Method

A structured approach to collect evidence and assess clinical practice in OUD related to chronic pain was defined based on a patient journey model describing care in a series of steps from patient initial engagement, through diagnosis to treatment. The patient journey model was defined by the authors based on their experience and with reference to other similar examples [24]. Sources describing clinical practice, such as national OUD and pain management guidelines, were identified by experts in relevant fields and collected. Data from sources were extracted and assessed by two reviewers familiar with analysis and the therapy area. Based on the results, a consensus on principles for clinical care to improve outcomes and future research were developed.

## Results

**Fig. 1:**
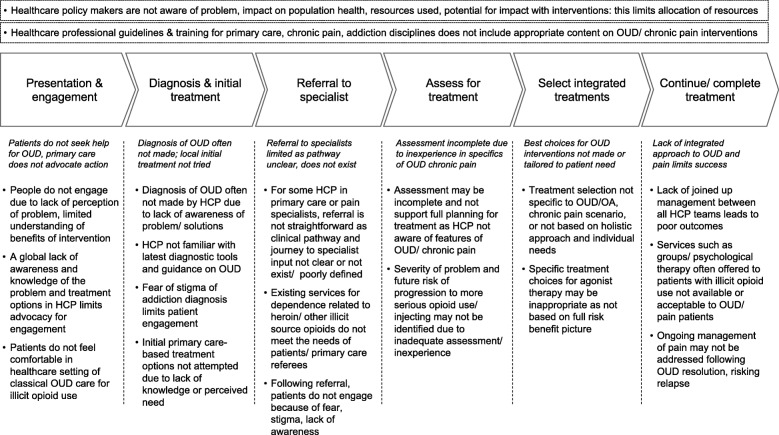
Current status of treatment, policy & practice. Treatment of OUD related to long-term opioid analgesic use and chronic pain

Care was assessed in Denmark, Finland, Iceland, Norway and Sweden. National treatment guidelines for the use of OA (e.g. in patients with chronic non-cancer pain or acute post-surgery treatment) were identified from professional societies or bodies responsible at national level for care, in Norway [25], Finland [26], Denmark [27], and Sweden [28]. National or similar guidelines for the management of OUD care in general were identified in Denmark [29], Finland [30], Norway [31], Sweden [32] and Iceland [33]. Specific guidance on the treatment of OUD related to chronic pain was identified in Sweden [32]. Limits to effective care were identified and are described according to the patient journey model (Fig. 1).

Structured assessment of the limits to successful care:

1. *Engagement*
  Patients or users of OA often do not engage with healthcare services to seek help for OUD related to chronic pain. This is due to a lack of awareness of the potential problems of long-term opioid therapy aiming to reduce symptoms associated with chronic pain from both patients and prescribing doctors, limited knowledge on the benefits of treatment, and limited engagement with the addiction services, which provide care mainly for patients with problems related to injection of illicit opioids and/ or psychoactive drugs, due to fear or stigma.
  Advocacy from healthcare professionals (HCP) in primary care (PC) and other settings may be insufficient in many cases, reducing the chance of patients agreeing to further steps towards diagnosis, referral and treatment. This lack of advocacy is often related to a low level of knowledge in PC of OUD in general and options for care specifically related to OUD and chronic pain.
2. *Diagnosis* in PC and addiction specialist settings is not optimal.
  Physicians, nurses and other HCP often have little training in the recognition and treatment of OUD. A lack of familiarity with appropriate diagnostic tools limits ability to effectively define patient problems and progress to appropriate care. Diagnosis may be difficult; for some patients it may be a challenge to accept that OUD is present, especially because the source of the dependence – OA pain medication – was prescribed and provided from within the medical system and is taken to reduce symptoms. Some pain relief is recognised upon taking the medication by the patient but less is recognised of side-effects such as the risk in escalating doses, developing dependence, cognitive impairment, gastrointestinal symptoms or other symptoms caused by opioids. Patients may avoid diagnosis despite evident problems due to a fear of attracting stigma when entering the conventional OUD treatment pathway.
3. *Referral pathways* are poorly defined or are inadequate.
  This limits the potential for progress with effective management. Care services developed for the management of addiction, related commonly to illicit opioid or drug use, often do not meet the needs of this specific population. The stigma associated with using existing, conventional treatment services limits participation of many patients after referral. HCP may be unaware of the risk of progression from OA use related to chronic pain to other forms of opioids and the risk of harm associated – limiting the urgency of referral.
4. *Treatment assessment* must be based on a full clinical picture and understanding of the needs of the patient.
  The goal of early intervention is important to prevent the gradual worsening of OUD and associated comorbidities over time. The difficulty in establishing a detailed clinical picture for each individual patient, including OUD, pain, psychiatric comorbidity (anxiety disorders and depression), and also in building relationships with patients due to limited engagement and inappropriate treatment service set-up often complicate adequate assessment. Success in treatment is limited due to lack of an integrated and holistic approach to care. Across the range of stakeholders who provide the necessary parts of care, a lack or insufficient awareness of the specific and different nature of treatment for OUD related to chronic pain make it hard to deliver an integrated program required for success.
5. *Treatment* success is limited if the approach is not specific to the needs of patients with OUD related to chronic pain.
  Treatment must be based on a holistic approach and individual needs. The chronic pain patients seek relief from is difficult to treat in terms of reduced pain independent of given treatment. When tapering of opioids HCP should consider the potential of opioids to induce hyperalgesia and the possible outcome of, in a part of the population, worsening pain and function in daily life. Many stakeholders are required for effective care and outcomes are not optimal if clinical services are not integrated and aligned. Inappropriate treatment choices regarding medication, duration and therapy may result from inexperience or uninformed decisions. Guidelines do not provide information to assist clinicians in the practicalities of building and delivering a treatment plan by means of an integrated team specialized in this particular setting. Functions including medical treatment, psychological and social therapy must be aligned for success – a lack of awareness and experience in treating patients in this population makes this more difficult to achieve. Disjointed management across different healthcare functions may lead to inadequate service delivery and poor outcomes.

*Overall* the lack of understanding of the size and nature of the epidemiology of OUD related to chronic pain limits the development and provision of care services. In policy, at national and regional level, there is often a lack of clarity on the commitment to provide appropriate resources to ensure the identification and treatment of OUD related to chronic pain.

## Discussion

**Fig. 2:**
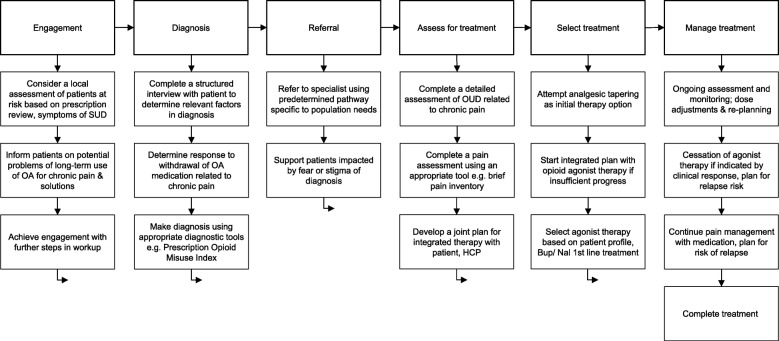
Suggested treatment approach, OUD related to chronic pain

There is potential to improve the approach for the management of OUD related to chronic pain. Current policy and practice does not reflect the specific needs of patients and available guidance does not provide sufficient direction on the practicalities of care overall. Guidance for clinical practice in pain management and/or OUD care may not be sufficiently useful beyond providing general information; it is recommended that such policy and guidance is developed further to meet the needs of the patients considering the comorbidity and challenges of treatment of this group. In relation to this, principles for best practice care of people with OUD related to chronic pain are described according to a patient journey model (Fig. 2).

### Increase engagement

Presentation and engagement with care – the starting point for treatment – can be made easier and more accessible. Awareness, advocacy and risk-based reviews of treatment are needed in services that are perceived as attractive, relevant and are specifically designed for the patient population [34].

1. In PC and with specialists in addiction medicine, provide educational programs aimed at increasing advocacy by building awareness of the problem, knowledge of referral pathways and successful interventions [35, 36].
2. Consider prescription data reviews to identify patients at risk, with a potential need for help, as in other locations including: Europe [37], UK [12], Denmark [38], US [36], Australia [23, 39].
3. Develop simple decision-support tools to help prescribers of OA, pharmacists and others to target, plan and hold discussions with patients about dependence problems, referral and treatment options [36, 39].
4. Set up and support the use of digital and other easy-to-access, publicly available tools (digital applications to self-assess, telephone and web helpline/ chat websites) to provide support and trusted resources for patients, carers, and family members [36, 40].

### Improve diagnosis

The gap in diagnosis and referral limits uptake of successful interventions and is based on limited awareness of proven tools and successful pathways to treatment.

1. Provide specific training for HCP in PC to improve skills and knowledge of diagnosis and referral pathways. Addiction and pain management specialists and national authorities should collaborate in providing educational pathways for HCP and psychiatrists. This includes drug screening and detection of relevant behavioural changes such as using opioids for reasons other than pain, to “get high” or “manage stress” [41], rapidly escalating demands for dose increases, unusual increase in doses, observed or reported intoxication or unexplained withdrawal symptoms, repeatedly reporting that opioid medication was lost, stolen, or destroyed; injection of opioids; threatening or harassing staff; repeatedly seeking prescriptions from other providers or emergency rooms; alteration, borrowing, stealing or selling prescriptions [42]; poor attendance at treatment review; appearing sedated at times; resisting drug screening; and deteriorating social function [21]. Education on modern diagnostic tools [36, 43, 44], access to training resources [45], practical guidelines for diagnosis [46] is recommended. Diagnostic and screening tools include those specific to OA or chronic pain (Current Opioid Misuse Measure [47], Prescription Opioid Misuse Index [44]). General tools may also be useful, including Leeds Dependence Questionnaire (LDQ) [48], Diagnostic and Statistical Manual of Mental Disorders, (DSM-5) [49], and International Statistical Classification of Diseases and Related Health Problems 10th Revision (ICD-10) [50].
2. Provide resources to set up and encourage the development of clinical practice aiming to achieve diagnosis in a joint effort between pain management specialists, addiction specialists and PC [46]. The model of care may be different in each location but the aim is co-operation with joint goals, plans and coordinated delivery of care with measurement of outcomes supported by patient registries. The ideal model of care presents a dedicated service for patients based on organisation of resources from PC, pain and addiction services.
3. Provide tools to assist HCP in working with patients to agree a “contract” defining goals of treatment and prescription renewal. If this contract cannot be maintained, this may be a point of acceptance of the need for help. Experience with a trial of tapering of prescribed opioids, performed in cooperation with the patient, is an important step in diagnosis; a lack of progress in dose tapering may indicate OUD.

### Increase the chance of successful referral to specialist care

Referrals to specialist care are limited because of unclear or inappropriate pathways.

1. Ensure at policy and clinical practice level that there is a referral pathway appropriate to the needs of patients with an option, when practical, to offer initial specialist consultation in PC to reduce stigma [40]. Patient with OUD related to chronic pain are more likely to engage, and be retained, in treatment if care pathways are provided that are distinct to the services offered to persons with OUD related to use and injection of illicit opioids [12, 51].

### Improve treatment assessment and choices

Non-optimal treatment assessment and choices of treatment may result from inexperience of HCP.

1. A specific clinical assessment is recommended for patients with OUD related to chronic pain [21, 46, 52]. A detailed profile or treatment inventory is required with status (e.g. pain, mental health status, anxiety, depression, other psychiatric disorders), severity of dependence and identification of other substances in use (non-opioid medications with addictive potential such as benzodiazepines, stimulants, alcohol/ nicotine and other substances). A pain assessment is required; if pain is a dominant feature, management led by pain specialists may be recommended. A biopsychosocial approach is useful in pain investigation to understand how pain interferes with life and to understand how long-term opioid use may contribute to suffering at individual level. Many tools to assess pain may be used: The Brief Pain Inventory (BPI) [53], The Client Health Questionnaire (PHQ-9) [54], EQ-5D-5 L [55] or a composition of tools as in Swedish Quality Registry for Pain Rehabilitation [56]. For patients with co-existing mental health problems, plans should be made to manage these issues. Severity of dependence guides decision-making and is the key for treatment assessment. Patients with severe problems may require higher doses of medication or need longer to achieve recovery. This may be assessed with tools such as DSM-5 [49] or ICD-10 [50].
2. A joint individualised [52, 57] treatment plan integrated across addiction services, pain clinics, psychiatry and PC services with a long-term view on care is required [46].
3. Management of existing OA medications for chronic pain and planning to reduce or taper medications over time in agreement with the patient [46]. Tapering can be performed with the prescribed drug or a switch to buprenorphine/ naloxone [58]. It may be possible to stop opioid medications and use adjunctive medication to support those agreeing to this plan, and not displaying signs of psychological dependence.
4. Treatment choices start with a managed reduction/ tapering of current OA, which can be tried either as a slow tapering in an outpatient setting, or faster in an inpatient setting. If these options are not successful, treatment with opioid agonist therapy (OAT) as part of an integrated psychosocial care program may be required [39, 46, 52]. Treatment should be planned on the basis of a detailed patient assessment or “inventory” [46, 52].
5. Treatment with OAT may be recommended if dose reduction/ tapering does not lead to improvement and OUD is considered to be moderate to severe. Based on the patient inventory including full listing of prescription and other drugs in use by the patient, a single dose of one opioid agonist can be prescribed. Commonly prescribed options include buprenorphine/ naloxone (as recommended in specific, existing national guidelines in Sweden), methadone or buprenorphine [52]. The decision to use OAT, and the exact treatment plan including choice of medication, is based on clinical scenario, risk profile, social & family situation, patient preference and assessment of safety and risk factors such as misuse, diversion, risk at home, concurrent addictive behaviour to other substances (e.g. alcohol or benzodiazepines), previous overdoses, psychiatric comorbidity [46].
6. Buprenorphine/ naloxone is a common initial recommendation if OAT medication is considered, in alignment with guidance [32]. Buprenorphine, because of potential for diversion, and methadone, due its profile with side effects including sedation and risk of overdose [45], may commonly be less attractive starting choices. No one choice of medical therapy is suitable for all patients; it is important to tailor therapy based on the needs of the individuals [52].
7. Intensive treatment monitoring is needed as standard [21], especially at the beginning of treatment programs [52] and strategies to respond in the event of relapse should be in place.
8. Adjustments of dose and choice of programs for psychosocial support may be necessary; decisions are guided by clinical progress including factors such as use of other opioids or addictive substances, craving and potentially other psychiatric symptoms such as anxiety or depression.
9. Cessation of OAT is guided by clinical response; many patients are expected to complete therapy and cease using any opioids. Duration of therapy may extend beyond 1 year [21]. Therapy should not be stopped prematurely and against the patient's will; therapy discontinuation should be carefully planned in discussion with patients and HCP jointly.
10. Access to appropriate specialist support groups specific to OUD and chronic pain problems for patients and their families should be available and actively referred to, for example behavioural therapy [12]. Therapy focused on acceptance of the problem is also recommended; this may include mindfulness coaching. Special education about the nature of chronic pain and support for patients is important; catastrophizing thoughts about the severity of pain and problems in relation to changing analgesic mediations are common in many patients. It is important to address this issue as it may be possible, at least in some part, to address it with education and development of coping strategies.

This analysis focuses on the steps of care specific to OUD related to chronic pain; it is important to also act to prevent problems emerging. It is important that all patients with chronic pain receive appropriate care. Where treatment with OA is considered, screening for risk of substance use disorder (SUD) in general is important in the evaluation of medication treatment of pain. Prescribing OA needs special attention for people at risk for SUD and special precautions are needed if OA are necessary. OA prescription practice should be well-founded to ensure appropriate pain care is provided for chronic pain patients – this in turn will limit initiation of large scale harmful opioid use. Early intervention is needed to avoid treatment with OA for pain developing into chronic OUD. An integrated and multidisciplinary service should be provided for all patients who develop OUD in relation to chronic pain. It is important to set up an environment in which there is an ongoing recognition of the specific needs of populations with OUD related to chronic pain, to design care services to meet the needs of these groups and to support HCP and other services in providing integrated and individualised care. It is important to define the ideal care set up at community and specialist levels, with goals of initial, short and longer-term treatment. In the longer-term perspective education to reduce the impact of stigma and related barriers to treatment may be valuable: starting with practical treatment steps can also be a part of changing this.

This work is based on an assessment of current approaches to OUD care in chronic pain setting with insights from specialists each with at least 10 years’ experience in OUD (10) and pain management (1); there can be benefit in including other pain specialists and organisations. This work is based on review of evidence from Nordic countries; this may limit wider applicability but the challenges and principles for managing OUD related to chronic pain identified here likely do however apply more widely. The existence of health registries and the similar approaches to OUD management and healthcare in general in Nordic countries present an opportunity to collect evidence to define the magnitude of the problem in greater detail, for example by assessing OA prescription data, and to develop further evidence-based approaches to clinical practice.

## Conclusion

The population with OUD related to chronic pain may be underserved for healthcare and find it hard to access therapy matched to their specific needs; there is an opportunity to improve the outcomes of treatment in these populations with specific care pathways. Principles for managing this complex problem are recommended here based on a consensus of clinical experience. Clinical practice experience highlights the need for policy change and specific practice development to make it possible to address the needs of this population; this includes the development of specific tailored care pathways. There is a need also to plan for research based on the opportunity in the region, provided by integrated health registries and common approaches to care, to define with increased resolution population needs and to confirm evidence supporting specific interventions. This is a call to action for health services and research organisations to act to improve outcomes for many with chronic pain and OUD.

## Abbreviations

HCP: Healthcare professional
OA: Opioid analgesics
OAT: Opioid agonist therapy
OUD: Opioid use disorder PC Primary care
SUD: Substance use disorder

## References

[1] Brat GA, Agniel D, Beam A, Yorkgitis B, Bicket M, Homer M (2018). Postsurgical prescriptions for opioid naive patients and association with overdose and misuse: retrospective cohort study. Bmj.

[2] National Academies of Sciences Engineering and Medicine; Health and Medicine Division; Board on Health Sciences Policy; Committee on Pain Management and Regulatory Strategies to Address Prescription Opioid Abuse (2017). Pain management and the opioid epidemic: balancing societal and individual benefits and risks of prescription opioid use.

[3] Vowles KE, McEntee ML, Julnes PS, Frohe T, Ney JP, Van Der Goes DN (2015). Rates of opioid misuse, abuse, and addiction in chronic pain: a systematic review and data synthesis. Pain.

[4] Manhapra A, Arias AJ, Ballantyne JC. The conundrum of opioid tapering in long-term opioid therapy for chronic pain: a commentary. Subst Abus. 2017:1–10. 10.1080/08897077.2017.1381663. 10.1080/08897077.2017.1381663 PMC6129223 28929914

[5] Chou R, Turner Judith A, Devine Emily B, Hansen Ryan N, Sullivan Sean D, Blazina I (2015). The effectiveness and risks of long-term opioid therapy for chronic pain: a systematic review for a national institutes of health pathways to prevention workshop. Ann Intern Med.

[6] Bedson J, Chen Y, Hayward RA, Ashworth J, Walters K, Dunn KM (2016). Trends in long-term opioid prescribing in primary care patients with musculoskeletal conditions: an observational database study. Pain.

[7] Edlund MJ, Martin BC, Russo JE, Devries A, Braden JB, Sullivan MD (2014). The role of opioid prescription in incident opioid abuse and dependence among individuals with chronic noncancer pain: the role of opioid prescription. Clin J Pain.

[8] Burkinshaw P, Knight J, Anders P, Eastwood B, Musto V, White M, et al. An evidence review of the outcomes that can be expected of drug misuse treatment in England. London: Public Health England; 2017. Available from: https://assets.publishing.service.gov.uk/government/uploads/system/uploads/attachment_data/file/586111/PHE_Evidence_review_of_drug_treatment_outcomes.pdf.

[9] Indivior UK Limited (2015). Summary of Product Characteristics: Subutex 0.4mg sublingual tablets.

[10] Indivior UK Limited (2015). summary of product characteristics: Suboxone tablets 8mg/2mg.

[11] Rosemont Pharmaceuticals limited (2014). Summary of Product Characteristics: Methadone Hydrochloride DTF 1mg/1ml oral Solution.

[12] Royal College of General Practitioners (2013). Prescription and over-the-counter medicines misuse and dependence.

[13] Fiellin DA, Schottenfeld RS, Cutter CJ, Moore BA, Barry DT, O’Connor PG (2014). Primary care–based buprenorphine taper vs maintenance therapy for prescription opioid dependence. JAMA Intern Med.

[14] Ballantyne JC, LaForge KS (2007). Opioid dependence and addiction during opioid treatment of chronic pain. Pain.

[15] Marr E, Hill D. Optimising service provision for prescribed opioid analgesic dependence. Heroin Addict Relat Clin Probl. 2015;17:13–7.

[16] Shipton EA, Shipton EE, Shipton AJ. A review of the opioid epidemic: what do we do about it? Pain Ther. 2018; 10.1007/s40122-018-0096-7. 10.1007/s40122-018-0096-7 PMC5993689 29623667

[17] Dowell D, Haegerich T, Chou R. CDC guideline for prescribing opioids for chronic pain - United States. Recomm Rep. 2016;65:1–49. https://doi.org/10.3109/15360288.2016.1173761. 10.15585/mmwr.rr6501e1 26987082

[18] British Medical Association (2017). Chronic pain : supporting safer prescribing of analgesics.

[19] The Royal Australian College of General Practitioners. Prescribing drugs of dependence in general practice, Part C2: The role of opioids in pain management. Melbourne: The Royal Australian College of General Practitioners; 2017. Available from: https://www.racgp.org.au/download/Documents/Guidelines/Opioid/Addictive-drugs-guide-C2.PDF

[20] Dematteis M, Auriacombe M, D’Agnone O, Somaini L, Szerman N, Littlewood R, et al. Recommendations for buprenorphine and methadone therapy in opioid use disorder: a European consensus. Expert Opin Pharmacother. 2017; 10.1080/14656566.2017.1409722. 10.1080/14656566.2017.1409722 29183228

[21] Clinical Guidelines on Drug Misuse and Dependence Update 2017 Independent Expert Working Group: Drug misuse and dependence: UK guidelines on clinical management. London: Department of Health. 2017.

[22] Ministerio de Sanidad Servicios Sociales e Igualdad. Prácticas seguras para el uso de opioides en pacientes con dolor crónico. Madrid; 2015. 10.1016/j.semerg.2015.12.007. 10.1016/j.semerg.2015.12.007 26831544

[23] Robert Ali et al.: Prescription Opioid Policy: Improving management of chronic non-malignant pain and prevention of problems associated with prescription opioid use 2009;Available from: https://www.ranzcp.org/Files/Resources/College_Statements/Practice_Guidelines/Chronic-non-malignant-pain-2009.aspx.

[24] Roncero C, Vega P, Martinez-Raga J, Torrens M (2017). Chronic hepatitis C and individuals with a history of injecting drugs in Spain: population assessment, challenges for successful treatment. Eur J Gastroenterol Hepatol.

[25] Helsedirektoratet: Bruk av opioider - Ved langvarige ikke-kreftrelaterte smerter 2014;Available from: https://www.beta.legeforeningen.no/contentassets/05dc17c74d5a4dfa87d9046d9ca5e48c/utkast-veileder-bruk-av-opioider-ved-langvarige-ikke-kreftrelaterte-smerter.pdf.

[26] Kalso E, Pennanen P, Paaskoski S, Pihlainen K, Meririnne E, Hermanson T (2009). Opioidit pitkäkestoisessa kivussa.

[27] Whærens EE, Hansen-Nord G, Kjøgx H, Højsted J, Faarvang KL, Kibsgaard K (2015). Udredning Og Behandling/ Rehabilitering Af Patienter Med Generaliserede Smerter I Bevægeapparatet.

[28] Läkemedelsverket (2017). Läkemedelsbehandling av långvarig smärta hos barn och vuxna - behandlingsrekommendationer.

[29] Sundhedsstyrelsen. Vejledning til læger, der behandler opioidafhængige patienter med substitutionsmedicin. Copenhagen; 2017. [cited 2017 Sep 4].Available from: https://www.sst.dk/da/sundhed-og-livsstil/narkotika/~/media/796D337DC66D4F72A8991141B88BD699.ashx

[30] Alho H, Aalto M, Eskola K, Jousilahti P, Kahila H, Kivitie-Kallio S (2012). Huumeongelmaisen hoito, Käypä hoito -suositus. Duodecim.

[31] Helsedirektoratet (2016). Behandling og rehabilitering av rusmiddelproblemer og avhengighet Nasjonal faglig retningslinje for behandling og rehabilitering av rusmiddelproblemer og avhengighet.

[32] Socialstyrelsen (2017). Nationella riktlinjer för vård och stöd vid missbruk och beroende.

[33] SAA National Center of Addiction Medicine: Ársrit Medferfarsvids Íslandi. 2016. Available from: https://saa.is/grein/arsrit-medferdarsvids-saa-2016-komid-ut/.

[34] The National Center on Addiction and Substance Abuse (2017). Ending the Opioid Crisis: A Practical Guide for State Policymakers.

[35] Substance Abuse and Mental Health Services Administration. Substance Misuse Prevention Media Campaigns. Cent Appl Prev Technol. 2017; Available from: https://www.samhsa.gov/capt/tools-learning-resources/prevention-media-campaigns. [cited 2017 Jul 20]

[36] Johns Hopkins Bloomberg School of Public Health (2015). The Prescription Opioid Epidemic: An Evidence- Based Approach.

[37] O’Brien T, Christrup LL, Drewes AM, Fallon MT, Kress HG, McQuay HJ (2017). European pain federation position paper on appropriate opioid use in chronic pain management. Eur J Pain (United Kingdom).

[38] Kushniruk A, Borycki E, Kuo M-H (2010). Advances in electronic health records in Denmark: from national strategy to effective healthcare system implementation. Acta Inform Medica.

[39] Kovitwanichkanont T, Day C (2017). Prescription opioid misuse and public health approach in Australia. Subst Use Misuse.

[40] British Medical Association (2017). Supporting individuals affected by prescribed drugs associated with dependence and withdrawal. Presribed Drug Depend.

[41] The Opioid Therapy for Chronic Pain Work. VA/DoD Clinical Practice Guideline for Opioid Therapy for Chronic Pain. Washington D. C; 2017. Available from: https://www.healthquality.va.gov/guidelines/Pain/cot/VADoDOTCPG022717.pdf

[42] The Management of Opioid Therapy for Chronic Pain Working Group (2010). VA/DoD clinical practice guideline Management of Opioid Therapy for chronic pain.

[43] Brady KT, McCauley JL, Back SE (2015). Prescription opioid misuse, abuse, and treatment in the United States: an update. Am J Psychiatry.

[44] Knisely JS, Wunsch MJ, Cropsey KL, Campbell ED (2008). Prescription Opioid Misuse Index: a brief questionnaire to assess misuse. J Subst Abuse Treat.

[45] British Columbia Node of the Canadian Research Initiative on Substance Misuse (2015). Together, we can do this. Strategies to address British Columbia’s prescription opioid crisis. Recommendations from the British Columbia node of the Canadian research initiative on substance misuse.

[46] Faculty of Pain Medicine: Royal College of Anaesthetists (2017). Identification and Treatment of Prescription Opioid Dependent Patients.

[47] Meltzer EC, Rybin D, Saitz R, Samet JH, Schwartz SL, Butler SF (2011). Identifying prescription opioid use disorder in primary care: diagnostic characteristics of the current opioid misuse measure (COMM). Pain.

[48] Raistrick D, Bradshaw J, Tober G, Weiner J, Allison J, Healey C (1994). Development of the Leeds dependence questionnaire (LDQ): a questionnaire to measure alcohol and opiate dependence in the context of a treatment evaluation package. Addiction.

[49] American Psychiatric Association. Diagnostic and statistical manual of mental disorders: DSM-5. 5th ed. Washington: American Psychiatric Association; 2013. https://doi.org/10.1176/appi.books.9780890425596.

[50] World Health Organization. International Statistical Classification of Diseases and Related Health Problems: ICD; 2010. Available from: http://apps.who.int/classifications/icd10/browse/2010/en#/. [cited 2018 May 15]

[51] Public Health England (2013). Commissioning treatment for dependence on prescription and over-the-counter medicines : a guide for NHS and local authority commissioners.

[52] Kraus M, Lintzeris N, Maier C, Savage S. Recommendations for the prevention, detection, treatment and management of prescription opioid analgesic Dependence: outcomes from the opioid analgesic dependence education Nexus (OPEN) meeting. Int J Ment Health Addict. 2015; 10.1007/s11469-015-9590-x. 10.1007/s11469-015-9590-x PMC4871907 27340378

[53] Atkinson TM, Mendoza TR, Sit L, Passik S, Scher HI, Cleeland C (1991). The brief pain inventory. Clin J Pain.

[54] Kroenke K, Spitzer RL, Williams JBW (2001). The PHQ-9: validity of a brief depression severity measure. J Gen Intern Med.

[55] Reenen M Van, Janssen B: EQ-5D-5L user guide. Rotterdam, 2015.Available from: https://euroqol.org/wp-content/uploads/2016/09/EQ-5D-5L_UserGuide_2015.pdf

[56] Nationella Registret över Smärtrehabilitering (2013). Manualer - “Lathund” med diagnoskriterier.

[57] Rindom H, Fink-Jensen A, Aaen-Larsen B, Thiesen H (2016). Opioider (iatrogen afhængighed).

[58] Baron M, McDonald P (2006). Significant pain reduction in chronic pain patients after detoxification from high-dose opioids. J Opioid Manag.

